# Maternal Dietary Patterns and Practices and Birth Weight in Northern Ghana

**DOI:** 10.1371/journal.pone.0162285

**Published:** 2016-09-09

**Authors:** Abdulai Abubakari, Albrecht Jahn

**Affiliations:** 1 Community Nutrition Department, School of Allied Health Sciences, University for Development Studies, Tamale, Ghana; 2 Institute of Public Health, Medical Faculty, University of Heidelberg, Heidelberg, Germany; The University of Manchester, UNITED KINGDOM

## Abstract

**Background:**

Adequate maternal nutrition is a key factor for achieving good pregnancy outcomes. Moreover, inadequate dietary intake during pregnancy is considered an important contributor to maternal malnutrition in developing countries. Although some studies have examined the effect of the entire diet on birth outcome, most studies have been very narrow because they considered the effect of single nutrient. The single nutrient approach is a major setback because usually several nutrient deficiencies are more likely to occur than single deficiencies especially in low-income settings.

**Objectives:**

The main aim of this study was to investigate the association between maternal dietary patterns, and practices and birth weight in Northern Ghana.

**Participant Settings:**

A facility-based cross-sectional survey was performed in two districts in the Northern Region of Ghana. The selected districts were the Tamale Metropolis and Savelugu-Nanton District. These districts were purposively sampled to represent a mix of urban, peri-urban and rural populations, therefore ensuring that the distribution in social groups of the study population was similar to the entire population of the region. In all, 578 mothers who were drawing antenatal and postnatal care services were interviewed using a questionnaire, which asked the mothers about their frequency of consumption of individual foods per week since they became pregnant or when they were pregnant.

**Statistical Analysis:**

We determined dietary patterns by applying a factor analysis with a varimax rotation using STATA. Multivariate analysis was used to establish association between maternal factors and dietary patterns. Logistic regression was used to assess the association between dietary practices and patterns and birth weight.

**Results:**

Women who ate outside the home twice a week (OR = 1.6 & 95% CI; 1.1–2.45, P; 0.017) and those who practiced ‘pica’ (OR = 1.7 & 95% CI; 1.16–2.75, P; 0.008) had increased odds for low birth. Two dietary patterns were identified—namely ‘health conscious’ and ‘non-health conscious’. Health conscious diet (OR = 0.23 95% CI 0.12–0.45 per standard deviation change in scores, P; <0.0001) and dietary diversity score (OR = 0.10 95% CI 0.04–0.13 per standard deviation change in scores, P; <0.0001) showed a protective effect for low birth weight respectively after adjusting for gestational age.

**Conclusion:**

Mothers who practiced good nutrition such as consuming foods across and within the various food groups were less likely to have low birth weight babies. Our findings buttress the importance of optimal nutrition during pregnancy.

## Introduction

Pregnancy is a critical period during which good maternal nutrition is key in ensuring successful pregnancy outcome. Nutritional needs are higher when pregnant and meeting these needs helps protect the long-term health of both fetus and mother. Pregnancy is a period of increased metabolic demands, with modifications in the woman's physiology and the requirements of a growing fetus [1]. There is much evidence supporting the link between inadequate maternal nutritional status and adverse pregnancy outcomes [2–4], poor infant survival [4, 5], risk of chronic diseases and impaired mental development in later life [6–10]. For instance it is found that deficiencies in intake of energy or specific nutrients during this ‘critical period’ may have adverse impact on health outcomes in later adult life [6, 11]. Evidence available also shows that women who consumed minimal amounts over the first eight-week period had a higher mortality or disorder rate concerning their offspring than women who ate regularly. This suggests that children born to well-fed mothers had a more favorable intrauterine environment [12].

Dietary intake during pregnancy has the potential to influence birth outcomes [9, 13]. Women of reproductive age, especially pregnant women, in low and middle income countries are at risk of several micronutrient deficiencies, such as iron, folic acid, iodine, zinc, vitamins A and D, riboflavin, B6 and B12, which could adversely affect the mother and pregnancy outcome [13, 14, 15]. More importantly, women living in developing countries are particularly at increased risk for malnutrition during pregnancy due to poverty, poor diet quality and quantity, high intensity of agricultural labor, and high fertility rate [16].

Although some studies have examined the effect of the entire diet on birth outcome, most studies have been very narrow because they considered the effect of single nutrient [3]. The single nutrient approach is a major setback because usually several nutrient deficiencies are more likely to occur than single deficiencies especially in low-income settings [17]. This is even more challenging to establish in populations where food is eaten from a common pot or where communal eating is practiced. In this situation it is difficult to quantify an individual intake [18]. Therefore, an indicator that could measure multiple nutrient deficiencies may be the best marker of the general adequacy of nutrient intake in these settings. Identification of dietary patterns and assessing dietary diversity has become important tool in nutrition epidemiology that could explore the relationship between dietary intake and health outcome [19].

Various dietary patterns have been identified by several studies, which could influence pregnancy outcome. For example, Thompson & co. [19] have described the relation between pattern of diet that a mother is consuming and the effect of this diet in relation to given birth to a small for gestational age (SGA) infant. In America researchers found that infants with mothers who practiced inappropriate nutrition had significantly lower weight-for-age Z-scores (WAZ) as well as lower head circumference Z-scores (HCZ) [20].

Moreover, it is suggested that dietary diversity scores could also be used as a proxy measure of micronutrient and diet adequacy of women [21, 22]. Increasing the variety of foods across and within food groups is recommended in most dietary guidelines internationally [22]. For instance, it is shown that intakes from a variety of foods within and across food groups are thought to improve intakes of variety of nutrients and promote good health [23]. Dietary diversity focused on overall dietary quality rather than on intakes of individual foods and nutrients [24, 25]. In Pakistan researchers concluded that dietary diversity is a good proxy indicator for nutritional status of pregnant women [26]. Evidence from Ghana also shows that individual dietary diversity scores is a significant predictor of mean birth weight and low birth weight [27].

Reports suggest that pregnant women using district and community health facilities may not necessarily have access to expert knowledge on maternal nutrition in Ghana. Therefore it is advocated by local experts that the development of more effective nutrition intervention is needed [28, 29] in order to ensure optimal maternal and infant nutrition.

Since dietary habits are often induced by culture and by the kind of food available, for consumption, investigating the characteristic of diet and practices therein within a study population may provide basis for future interventions to improve maternal and infant nutrition. Therefore the main aim of this study was to investigate maternal dietary patterns and practices in relation to birth weight in Northern Ghana. Specifically the study investigated; (1) The association between dietary practices and low birth weight; (2) Dietary patterns and associated factors during pregnancy; (3) The association between dietary patterns and low birth weight.

## Material and Methods

### Study design

The study design including the methods used to generate data on demographic and socio-economic factors as well as antenatal records used in the present study have been described previously and published [30]. The study took place in two districts in the Northern Region of Ghana. The region is among the poorest regions in the country. Agriculture and related activities are the main occupation of the people in the region. The region has 26 districts, with 24 of them being predominantly rural [31]. This notwithstanding, about half of the population live in urban areas with Tamale Metropolis, the regional capital being the most urbanized city in the region. The selected districts were Tamale Metropolis and Savelugu-Nanton District. Tamale Metropolis is predominantly urban and Savelugu Nanton District is predominantly rural. These districts were purposively sampled to represent a mix of urban, peri-urban and rural populations, therefore ensuring that the distribution in social groups of the study population was similar to the entire population of the Northern Region. A fairly mixed population is also necessary because of the effect of the double burden of malnutrition phenomenon where undernutrition and overnutrition may co-exist.

### Study population and sampling

A longitudinal component was conducted at the Tamale Teaching Hospital. The target group was pregnant women who were in the second trimester of their pregnancy and attending antenatal clinic (ANC) at the Tamale Teaching Hospital. Only singleton pregnancy was considered. Two hundred and sixteen (216) pregnant women were selected using consecutive sampling technique and those who agreed to participate in the study were asked to sign a consent form. They were followed until delivery for data that is not relevant to the present analysis.

The 2016 was arrived at using the following expression;

$$\text{N}={\left({\text{Z}}_{1}{}_{-\alpha }\right)}^{2}\;(\text{P}\;\left(1-\text{P}\right)/{\text{D}}^{2})$$ (1)

[32]

Where Z_1-α_ = Z_0.95_ = 1.96 (from normal distribution table for CI of 95%)

The prevalence of low birth weight (P) at the Tamale Teaching Hospital was 16% = 0.16

And the degree of precision for this study was estimated at 5% implying that the prevalence of abnormal birth weight babies at the Hospital will be between 11–21%.

This implies that N = (1.96^2^) (0.16 (1–0.16)/0.05^2^) = 206. We assumed 5% lost to follow up, hence the actual number of women required to estimate the prevalence of low birth weight between 11–21% was 206/0.95 = 216.

Therefore, the total number of pregnant women to be studied at the Tamale Teaching Hospital (TTH) was 216.

The 216 pregnant women were selected based on the following; the pregnancy was in the second trimester; the pregnant woman was not severely ill and regular ANC attendance. Consecutive sampling procedure was used to select the pregnant women. This implied that all pregnant women who met the criteria enumerated above were selected per every visit we made to the Hospital until all the 216 women were selected.

The study also targeted mothers receiving postnatal and child welfare clinic services and have babies who were ≤ 40 days old. This was because we needed to take pregnancy records from the antenatal booklet, which is only available during the postnatal visit after which child welfare book is used.

Three hundred and eighty four (384) mothers were approached and those who agreed to participate in the study were asked to sign a consent form. The sample size was determined using an estimated low birth weight prevalence of 50% for the entire Northern Region since the prevalence of Low birth weight in the entire region was not known. Therefore, the prevalence of low birth weight in the four hospitals/in the Northern Region was estimated to be between 45–55% since the precision and the confidence interval remained 5% and 95% respectively.

Hence the sample size for the cross sectional component of this study was determined as below;

Using Eq 1, implies that N = (1.96^2^) (0.5 (1–0.5)/0.05^2^) = 384

This means that a total of 384 mothers were sampled.

In all 96 women were selected from each of the four hospitals, which included Tamale Teaching Hospital (the largest referral hospital in Northern Ghana), Tamale Central, Tamale West and Savelugu District Hospital using consecutive sampling technique.

Therefore, in all 600 subjects were approached and recruited for the entire study but 216 of them were pregnant mothers attending antenatal clinics at the TTH while the remaining 384 were mothers with babies between 0 and 1 months old.

### Data collection

Structured questionnaires were administered to the mothers by a team of interviewers. The questionnaires collected information on demographic and socio-economic characteristics of mothers. Information on health status, weight at first antenatal and weight per antenatal Care (ANC) visit, height of mother and birth weight were retrospectively recorded from the ANC booklet for the nursing mothers where as the remaining information (weight and gestational age per ANC visit, date of delivery and weight of infants) of the pregnant women were recorded as and when they came for ANC visit until delivery.

Food frequency questionnaire were administered to the mothers within the first month after delivery while the pregnant women completed the questionnaire in the second trimester. The food frequency questionnaire used was identical to and derived from Nti [33] and GDHS 2008 [34], which have been validated and used in Ghana. The FFQ asked the mothers about their frequency of consumption of individual foods per week since they became pregnant or when they were pregnant. Questions were asked about individual foods in the broad categories: fresh fruits; vegetables; rice/noodles; chicken, meat or organ meat, dairy foods/milk, cereals, cakes and biscuits; sauces/soups and snacks, roots and tubers; yam, cassava, cocoa yam and potatoes, legumes; beans, Bambara beans, and cowpea and takeaways; drinks and fast foods. The questions on each food were asked in the following manner; how often have you on average had the following since you became pregnant/when you were pregnant. The choices were (1) >6 times a week, (2) 5 times a week, (3) 4 times a week, (4) 3 times a week, (5) 2 times a week, (6) once a week (7) 3 times a month (8) 2 times a month (9) once a month (10) not at all. The questionnaire collected information on 55 food items but only 36 foods were considered in the analysis because the rest (19 foods) were rarely consumed and for that matter had correlation of zero.

The data was entered using Epi Info (version 3.4.1, 2005, Center for Disease Controle) and later imported to STATA (Version12.1, 2012, statacorp) for analysis. Consistency and plausibility checks were done after the data entry to ensure that errors were reduced.

### Statistical analysis

Descriptive statistics including percentages and frequencies were computed for dietary practices such as ‘out of home eating’, ‘pica’ practices (consumption of rocks and clay during pregnancy), skipping of meals and pregnancy related conditions such as nausea and vomiting.

The analysis to determine characteristics of dietary patterns was carried out using all the 36 food items from the questionnaire. Dietary patterns can be derived a priori, where foods are classified into patterns based on their known functions and effects on health, or a posteriori, where statistical methods such as factor analysis or principal component analysis are used to categorize foods that are usually eaten together into groups [35]. Both methods have been found to be biologically meaningful [36, 37].

In this analysis we determined dietary patterns using factor analysis with a varimax rotation. Variance was based on rotated sums of squared loading and the varimax with Keiser Normalization was used as the orthogonal rotation method, as it maximizes the loading of each variable on one of the extracted factors whilst minimizing the loading on all other factors.

Using a varimax rotation ensured that the distributions of the scores were centered around 0, with a standard deviation of 1. This allows easy interpretation of the results, as the point estimates from the regression analyses will be equivalent to a change of the magnitude of the corresponding number of standard deviations [19]. The factors were obtained using scree plots and the variance explained by each factor [19, 21, 38]. Across dietary patterns the distribution of loading was assessed to be sure that there was no intersection between factors. Factor loadings, which had a magnitude of ≥0.3 [19] was considered to have a strong association and the foods within a factor with loadings of these values were considered to be descriptive of the ‘pattern’ of diet associated with this factor. A score was then generated for each of the factors. Factor 1, which had higher scores for vegetables, fruits, food prepared out of cereals, legumes, roots and tubers and negative scores for high sugar/energy snacks (such as fan choco, fan ice, fan milk and sweets) was described as ‘health conscious’ diet while factor 2, which had higher loading/scores for high sugar/energy snacks and low scores for vegetables, fruits, cereals roots and tubers was described as ‘non-health conscious’ diet.

Prior to the analysis the Kaiser-Meyer-Olkin measure of sample adequacy, which is prerequisite for PCA or factor analysis, was performed and found to be in an acceptable range (0.756). Besides this, Bartlett’s test of sphericity performed showed no evidence of identity of correlation matrix in the data and for that matter the data set was considered appropriate for factor analysis (Prob>chi2 = 0.0000).

Dietary quality of the women was determined using women dietary diversity score (WDDS) [22]. The potential score of the WDDS is 0–9, which represents the likelihood of micronutrient adequacy of the diet, and therefore food groups included in the score are meant for this purpose [22]. According to FAO [22] the food groups included in the WDDS include starchy staples, dark green leafy vegetables, other vitamin A rich fruits and vegetables, other fruits and vegetables, organ meat, meat and fish, eggs, legumes nuts and seeds, milk and milk products. However, this was customized based on Ghanaian staples. Dietary diversity scores were obtained qualitatively using food frequency questionnaires, which were designed to capture information over a period of one week. The dietary diversity score was obtained by simple adding the food groups.

Classification of weight gain during pregnancy was based on pre-pregnancy BMI and according to IOM recommendations [39, 40]. Details on the calculation and classification of weight gain during pregnancy according to IOM recommendations and BMI have been described and published previously [30].

The socio-economic status of the household was determined using the household wealth index as a proxy, which was derived from household assets such as availability of potable water, electricity, television, refrigerator, motorcycle, car/tractor/truck, washing machine, gas cooker and livestock. The calculation of wealth index has also been described and published previously [30].

According to the world health Organization [41], a low birth weight infant is one born with birth weight <2.5kg. Macrosomia was defined as birth weight greater than or equal to 4.0kg. Therefore, this study considered all birth weights greater than or equal to 4.0kg as macrosomic births. Birth weight ≥2.5kg< 4.0kg was considered normal.

Associations between maternal and infant characteristics and mean women dietary diversity score were determined by independent *t*-test. One-way ANOVA was used to compare means where more than two categories were formed. Multiple testing was controlled for by the use of Bonferroni corrections. The associations between dietary patterns and socio-demographic and life style factors of mothers were determined using multivariate regression.

To determine the factors influencing the mother’s choice of a particular dietary pattern a multivariate analysis was done. The mother’s factor score for each dietary pattern (health conscious and non health conscious diet) were used as dependent variables. A Logistic regression was used to establish association between dietary practices, and patterns and birth weight. A p-value<0.05 at 95% confident level was considered statistically significant.

The mothers were asked to sign a written consent form. For those who could not read and write, this was done through an interpreter. The guardians or parents were asked to sign a written parental or guardian form on behalf of the minors while the minors were asked to sign an assent form. The Ethics committees of Navarongo Health Research Center (Ref. No: App/MatNut/01/2014) and the University of Heidelberg institutional review board approved this study.

## Results

### Socio-demographic characteristics of study participant

The study took place between February and August 2014. In all, 578 out of 600 women completed the study questionnaire representing a response rate of 96.0%. The study participants were mostly married women and predominantly Dagombas (the largest ethnic group in Northern Region). They were predominantly Muslims (94.29%). About 60% of the women had some formal education while 40% had no formal education. Also, about 77% of the respondents worked in the formal/informal sector but 23% of them were either house wives/students. Moreover, about 78% of the women we interviewed were urban dwellers where as 22% were rural dwellers. More to the point, 60% of the mothers had 1–2 babies while the rest had 3 or more babies.

The socio-economic status of the household was divided in to three categories. About 33% of the respondents were in the lower socio economic class, 34% in the middle class while about 32% were in the upper socio-economic class (Table 1).

**Table 1:** Maternal and household characteristics.

Variable	Categories	Frequency (No.)	Percentage (%)
Education	No formal education	231	40.0
	Any formal education	347	60.0
Mothers occupation	Informal	347	60.0
	Housewife	134	23.2
	Formal	80	13.8
Location	Urban	452	78.20
	Rural	126	21.8
Parity	Below Average (1–2)	350	60.5
	Average and above (≥3)	228	39.5
Age of Mother	16–20	15	2.6
	21–30	552	95.5
	>30	11	1.9
Socio economic status	Lowest quintile	193	33.4
	Middle quintile	197	34.1
	Upper quintile	188	32.5
Ethnicity	Dagomba	461	79.8
	Gonja	24	4.2
	Mampurisi	16	2.7
	Other ethnic groups	77	13.3
Religion	Muslim	545	94.3
	Christian	33	5.7
Household size	0–5	200	34.6
	6–10	245	42.4
	>10	133	23.0

### Dietary patterns

The data analysis revealed that two distinct dietary patterns best explained the dietary habits of women in this study. For descriptive purposes, they are labeled as ‘Health conscious’ and ‘non-health conscious’. The list of foods and the loading factors are shown in Table 2. Out of the 36 foods included in the analysis, 29 foods had loading factors greater than or equal 0.3 for at least one of the two dietary patterns. Foods that were classified as being characteristic of the non-health conscious diet included fan yogo (sweetened beverage), fan ice (ice cream), fan choco (chocolate energy drink), fan milk and alvaro (a local soft drink). The health conscious diet included TZ (a local dish made from corn flour), rice, kenke (local dish made from fermented corn dough), Banku (local dish made from a mixture of corn and cassava dough), yam, fruits (watermelon, mango, apples, avocado, banana, pawpaw, pineapple), traditional and exotic vegetables (carrot, tomatoes, dark green leafy vegetables, cabbage, salad, cucumber), meat, water, and eggs. The ‘health conscious diet’ has negative loading for fan choco, fan milk, fan ice, yogurt drink and sweets (Table 3).

**Table 2:** Loading factors from factor analysis using varimax rotation.

Variables	Health conscious diet	Non-health conscious diet
Percentage of variance explained	4.4%	3.0%
TZ^1^	0.4151*	-0.1339
Banku^2^	0.4731*	0.0392
Kenke^3^	0.5544*	-0.0969
Fufu^4^	0.5415*	-0.0177
Rice and Beans	0.4900*	0.0454
Jolof and fried rice	0.4638*	-0.00792
Soft drinks	0.0861	0.0005
Fan Choco (chocolate based beverage)	-0.0178	0.6749*
Fan milk (milk based beverage)	-0.0117	0.6229*
Fan ice (ice cream)	-0.0333	0.6702*
Fan yogurt	0.0712	0.4437*
Yogurt drink	-0.0852	0.4306*
Milk	0.1185	0.1776
Meat	0.0982	0.1666
Water	0.3460*	0.1680
Watermelon	0.5842*	-0.0402
Pineapple	0.4956*	0.1402
Apple	0.3291*	0.1070
Orange	0.1088	0.3762*
Mango	0.4259*	0.0215
Banana	0.4369*	0.0422
Avocado	0.4542*	0.0285
Traditional vegetables	0.4508*	-0.2670
Exotic vegetables	0.4102*	0.2865
Bread	0.1063	0.0423
Biscuit	0.0878	0.0952
Chocolate	0.0677	0.1695
Mashed Kenke Drink	0.3097*	0.1455
Tea	0.1926	0.0070
Coffee	0.0384	-0.0588
Pawpaw	0.4954*	-0.0796
Boiled and Fried yam	0.5431*	-0.0725
Fish	0.1281	0.3112*
Sweets	-0.0041	0.0920
Alvaro	0.1370	0.4561*
Fats and oil	0.5579*	0.1303

^1^ TZ (Tueozafi): made from corn and cassava flour,

^2^ Banku: made from fermented corn and cassava dough,

^3^ Kenke: made from fermented corn dough,

^4^ Fufu: made from pounded boiled yam and cassava.

* = Factor loading ≥0.3

**Table 3:** Maternal socio-economic factors and women dietary diversity scores.

Variable	Categories	Mean ± SD (DDS)	p-value
Age of mother/years	21–30	4.30±1.56	
	15–20	4.40±1.62	0.813^a^^,^ ^b^
	>30	4.32±1.65	
Mother’s location	Urban	4.42±0.10	0. 000^t^
	Rural	3.84±0.14	
Mother’s educational status	Had some formal education	4.34±1.63	0.387^t^
	Had no formal education	4.22±1.61	
Mother’s occupation	Informal	4.28±1.62	
	House wife	4.25±1.62	0.756^a^^,^^c^
	Formal sector	4.41±1.64	
Household socio-economic status	Middle class	4.38±1.49	
	Low class	4.21±1.68	0.587^a^^,^^d^
	Upper class	4.30±1.70	
Pica practice	No	4.32±0.10	0.311^t^
	Yes	4.17±0.14	
Out of home eating	No	4.33±0.12	0.347^t^
	Yes	4.19±0.10	
Ethnicity	Dagomba	4.30±1.60	
	Gonja	4.13±2.10	0.401^a^^,^^e^
	Mamprusi	4.0±1.93	
	Other ethnic groups	4.57±1.66	
Household size	0–5	4.39±1.73	
	6–10	4.3±1.54	0.401^a^^,^^e^
	>10	4.16±1.0	
Marital status	Married	3.0±1.20	0.065
	Co-habiting	4.32±1.61	

^a^ One way anova,

^t^ independent t-test,

^b^ 16–20, 21–30, >30,

^c^ Informal sector, Housewife, Formal sector,

^d^ Low income Household, Middle income household, Upper income household,

^e^ Dagomba, Gonja, Mampurisi, Other ethnicity,

^f^ 0–5, 6–10, >10

### Association between women dietary diversity and maternal characteristics

Among the respondents the mean dietary diversity score was 4.3±1.6. Higher mean dietary diversity score that was significantly different was observed in mothers who live in urban areas (0.6) as compared to those who live in rural areas. Although, there were slight differences in absolute terms within the other variables they were statistical not insignificant (Table 3).

In addition to this, higher dietary diversity score that were statistically significant were observed among mothers who had normal hemoglobin level (≥10.5 g/dL) (0.5), gained excessive weight during pregnancy (0.1), were overweight (0.6), were obese (0.8) and mothers who had macrosomic births (Birth weight ≥ 4.0kg). On the other hand, lower dietary diversification was observed in mothers who had low gestational weight gain (-1.0), were underweight before pregnancy (-1.3) and those who had low birth weight infants (2.8) (Table 4).

**Table 4:** Maternal and infant characteristics and dietary diversity scores (DDS).

Variables	Categories	DDS		
		N(%)	Mean ± SD	p-value
Weight gain according to IOM	Adequate weight gain	178(30.8)	4.9 ± 1.4	
	Low weight gain	361(62.5)	3.9±1.6	<0.0001^a^^,^^b^
	Excess weight gain	39(6.7)	5.0±1.6	
Pre-pregnancy BMI (kg/m^2^)	Normal (18.5–24.99)	334(57.8)	4.10±1.5	<0. 0001^a^^,^ ^c^
	Under weight (<18.5)	20(3.46)	2.7±1.6	
	Over weight (>25<30)	147(25.4)	4.7±1.5	
	Obese (>30)	77(13.3)	4.8±1.7	
Haemoglobin level (g/dL)	Below normal<11.0)	281(48.6)	4.0±0.1	0.039^t^
	Normal (≥11.0)	297(51.4)	4.5±0.1	
Gestational age in weeks	<37	140(24.2)	4.1±1.7	0.064^t^
	≥37	438(75.8)	4.4±1.6	
Birth weight (kg)	Normal (2.5–3.99)	356(61.6)	5.0±1.0	
	Low birth weight (<2.5)	162(28.0)	2.2±1.0	<0.0001^a^^,^ ^d^
	Too heavy (≥4)	60(10.4)	6.0±1.0	

^a^ One way anova,

^t^ independent t-test,

^b^ Inadequate, Normal excessive weight gain,

^c^ <18.5, 18.5–24.99, >25<30, >30,

^d^ Normal, Low birth weight, Too heavy

### Association between dietary pattern and socio-demographic and lifestyle factors

Factors that could potentially influence the mother’s choice of a particular dietary pattern were also analyzed. ‘Pica’ practice (95%CI; 0.1–0.5, P; 0.006) and upper socio-economic class status (95%CI; 0.1–0.4, P; 0.034) had higher loading respectively for non-health conscious diet. However, mothers who had no any formal education (95%CI; -0.4-(-0.1, P; 0.007), avoid both fish and meat (95%CI; -1.1-(-0.0), P; 0.043) during pregnancy had significantly lower loading respectively for non-health conscious diet (Table 5).

**Table 5:** Determinants of maternal dietary patterns/habits.

Variable	Measurements	Health conscious diet		Non-Health conscious diet	
		Coef (95%CI)	P-value	Coef (95%CI)	P-value
Out of home eating	No	Ref.			
	Yes	-0.2 (-0.4-(-0.1)	0.003	-0.0 (-0.2-(0.2)	0.765
Wealth index	Middle	Ref.			
	Low	-0. 1(-0.3–0.0)	0.159	-0.2 (-0.4-(-0.0)	0.030
	Upper	0.0(-0.2–0.2)	0.991	0.2 (0.0–0.4)	0.034
Education	Formal education	Ref.			
	No formal education	0.0 (-0.1–0.2)	0.821	-0.2 (-0.4–0.1)	0.007
Mother’s Occupation	Formal	Ref			
	Housewife/Student	-0.0 (-0.2–0.2)	0.939	-0.2 (-0.4–0.1)	0.182
	Informal	-0.0 (-0.3–0.2)	0.789	-0.2 (-0.4–0.1)	0.298
Description of diet during pregnancy	Eat meat and fish	Ref.			
	Avoid fish but eat meat	-0.1 (-0.4–0.2)	0.613	-0.3 (-0.7–0.1)	0.095
	Avoid meat but eat fish	0.3 (0.1–0.1)	0.009	-0.2 (-0.5–0.1)	0.129
	Avoid meat and fish	-0.1 (-0.5–0.4)	0.699	-0.8 (-1.1-(-0.0)	0.043
Location	Urban	ref			
	Rural	-0.1 (-0.2–0.1)	0.494	-0.1 (-0.3–0.1)	0.212
Age of mother	21–30	Ref.			
	15–20	0.125 (-0.1–0.3)	0.269	0.1 (-0.2–0.4)	0.389
	>30	-0.0 (-0.2–0.2)	0.979	-0.0 (-0.2–0.1)	0.646
Experienced nausea	No	Ref.			
	Yes	-0.1 (-6-0.8)	0.799	0.1 (-0.6–0.8)	0.811
Experienced vomiting	No	Ref.			
	Yes	-0.013 (-0.5–0.6)	0.865	0.0 (-0.2–0.2)	0.719
Eat more or less when nauseating/vomiting	Eat normal	Ref.			
	Eat less	-0.0 (-0.6–0.6)	0.950	0.1 (-0.6–0.8)	0.776
	Eat more	-0.2 (-0.5–0.9)	0.572	0.1 (-0.8–0.9)	0.872
Practiced pica	No	ref			
	Yes	0.0 (-0.1–0.2)	0.793	0.3 (-0.1–0.5)	0.006
Skip break fast	No	ref			
	Yes	-0.4 (-0.6-(-0.4)	<0.001	0.0 (-0.2–0.3)	0.839
Marital status	Yes	ref			
	No	0.5 (-0.2–1.2)	0.129	0.4 (-0.5–1.2)	0.427
Body mass index (BMI)	Normal	ref			
	Underweight	-0.4 (-0.7–0.0)	0.036	-0.0 (-0.4–0.4)	0.979
	Overweight	0.2(0.0–0.3)	0.025	0.1 (-0.1–0.3)	0.379
	Obese	0.1 (-0.1–0.3)	0.426	0.2 (-0.1–0.5)	0.156
Weight gain according to IOM	Adequate	Ref			
	Low	-0.2 (-0.3–0.0)	0.019	0.1 (-0.1–0.3)	0.364
	Excess	-0.1 (-0.4–0.2)	0.577	-0.1 (-0.4–0.3)	0.748

Ref: reference

On the other hand, mothers who avoid meat but eat fish (95%CI; 0.1–0.5, P; 0.009) and women who were overweight (95%CI; 0.2–0.3, P; 0.025) had significantly higher loading respectively for health conscious diet where as those who practice out of home eating (95%CI; -0.2-(-0.2), P; 0.003) during pregnancy period, skip breakfast (95%CI; -0.6-(-0.2, P; <0.001) and were underweight (95%CI; -0.7-(-0.0), P; 0.036) had significantly lower loading respectively for health conscious diet (Table 5).

### Association between dietary practices during pregnancy and birth weight

It was observed that 29.6% of the mothers practice ‘out of home eating’ (eating >2x/week from food venders and fast food restaurants). A majority (51.9%) reported eating less than usual during pregnancy while 45% ate normal and the remaining 3.1% reported eating more than usual. Moreover, the prevalence of ‘pica’ (craving for clay and rocks) practice was 22.8%. Also 87.8% of the respondents reported skipping breakfast while the remaining (12.1%) had breakfast on the regular basis during pregnancy period (Table 6).

**Table 6:** Association between dietary practices and birth weight.

Maternal dietary practices	Measures	N (%)	OR (CI 95%)	P-values
Out of home eating	No	408(70.4%)	Ref	
	Yes	178(29.6%)	1.63(1.1–2.45)	0.017
Food intake before and during pregnancy	Eat the same as before pregnancy	260(50.0%)	Ref	
	Eat less during pregnancy	300(51.9%)	0.77(0.53–1.12)	0.169
	Eat more during pregnancy	18(3.1%)	0.12(0.02–0.90)	0.039
Pica practice	No	446(77.1%)	Ref	
	Yes	132(22.8%)	1.79(1.16–2.75)	0.008
Description of diet	Eat both meat and fish	489 (84.6%)	Ref	
	Avoid fish but eat meat	27(4.8%)	1.34(0.51–3.53)	0.553
	Avoid meat but eat fish	49 (8.5%)	0.56(0.27–1.19)	0.133
	Avoid both	13(2.2%)	0.38(0.12–1.19)	0.098
Skip break fast	No	70(12.1)	Ref.	
	Yes	508(87.8%)	0.80(0.46–1.40)	0.432

Ref: reference

Model Power (Pseudo R^2^) = 0.31

In addition to this, women who ate outside the home during pregnancy were 1.6 times more likely to give birth to low birth weight infant than those who ate regularly at home during pregnancy (95% CI; 1.1–2.45, P; 0.017). In the same vein, those who practice ‘pica’ during pregnancy were 1.7 times more likely to give birth to a low birth weight baby as compare to those who did not practice pica (95% CI; 1.16–2.75, P; 0.008). Mothers who consumed more food (increased food intake compare to pre-conception period) during pregnancy period were 88% less likely to give birth to a low birth weight infants than those who ate the same as before pregnancy (95% CI; 0.02–0.90, P; 0.039 (Table 6).

### Association between maternal dietary pattern and birth weight

In the logistics regression model of low birth weight; at the univariate level, ‘health conscious diet’ (OR = 0.10; 95% CI; 0.10–0.20 per standard deviation change in scores, P; <0.0001) and dietary diversity score (OR = 0.1 95% CI; 0.04–0.12 per standard deviation change in scores, P; <0.0001) showed a protective effect for low birth weight respectively. The same trend was observed in the multiple logistic regressions adjusted by gestational age. The health conscious diet showed a reduced risk of LWB (OR = 0.23 95% CI; 0.12–0.45, P; <0.0001) per standard deviation change in scores) as well as dietary diversity scores (OR = 0.10 95% CI 0.04–0.13 per standard deviation change in scores, P; <0.0001) (Table 7).

**Table 7:** Association between dietary Patterns and birth weight.

Maternal dietary patterns	Bivariate		Multiple logistic regression (R^2^ = 0.78)	
	OR (CI 95%)	P-values	OR (CI 95%)	P-values
Health conscious diet	0.11 (0.10–0.20)	<0.0001	0.23 (0.12–0.45)	<0.0001
Non-Health conscious diet	1.0 (1.18–1.2)	0.834	1.04 (0.65–1.67)	0.956
Dietary diversity score	0.1 (0.04–0.12)	<0.0001	0.10 (0.04–0.13)	<0.0001

## Discussion

We have defined two dietary patterns and also demonstrated the relationship between pattern of diet that a mother is consuming and how it is associated with birth weight. Health conscious diet and dietary diversity were associated with decreased odds for low birth weight. Also, women who gave birth to low birth weight infant had significantly low mean dietary diversity scores.

We found that mothers who ate out of home and those who practice ‘pica’ during pregnancy have increased odds for low birth weight while those who reported that they consumed more food during pregnancy than before conception have decreased odds for low birth weight.

Beside this, factors that influence dietary patterns were also identified. For instance skipping breakfast, out of home eating and underweight were associated with low factor loading for health conscious diet while eating fish but not meat and overweight were associated with high factor loading for health conscious diet. On the other hand, pica practice and upper socio economic class were also associated with high factor loading for non-health conscious diet while low socio economic class, no any formal education and avoiding eating both meat and fish during pregnancy were associated with low factor loading for non-health conscious diet.

Many studies have shown that there exist a link between pica and maternal anemia but in contrast to this study they found no association between pica and mean birth weight [42, 43]. Others have found significant association between pica practice and low head circumference and maternal hemoglobin [44]. Ahmad and Co. [45] also shows that low maternal hemoglobin is significantly associated with low birth weight. This indirectly explains the observation made in this study. Out of home eating, which is associated with the consumption of non-hygienic, and junk foods especially in developing countries [46] was also associated with increased risk of low birth weight.

It has been observed that until recently, there have been few studies on dietary patterns of mothers during pregnancy [19] especially in Sub-Sahara Africa. The few studies that have been conducted on this topic among women observed varied number of patterns. For example a study in HIV infected pregnant women in Malawi identified three dietary patterns namely; ‘animal-based’, ‘plant-based’ and ‘grain based’ [47]. Another study conducted in HIV infected population in South Africa also identified four dietary patterns described as; ‘animal-based’, ‘stapled-based’, ‘recommended diet’, ‘egg-and breakfast-cereals’ and ‘legumes-and-vegetables’ [48]. Further, a study conducted in Mexican-American women [49] and in Finland [50] defined seven dietary patterns respectively. However, it is noted that in these studies lower factor loading of 0.2 was chosen instead of 0.3, which is used by others [19] including the present study. Also in Spain a study conducted among women from preconception to 6-months postnatal described two dietary patterns, namely; ‘sweetened beverages and sugar’ and ‘vegetable and meat’ [51], which is similar to the number of patterns identified in the current study. Another study by Thompson & co [19] among pregnant women in New Zealand identified three patterns namely ‘traditional’, ‘fusion’ and ‘junk’ diets. They found no difference in dietary pattern between early and late pregnancy because the distribution of dietary scores in early and late pregnancy did not vary.

Most of the studies conducted on maternal dietary patterns demonstrated the relationship between the patterns identified and birth weight, which is consistent with this study. For instance, the study conducted among Mexican-American women showed that nutrient dense and protein rich foods were associated with an increased infant birth weight whereas a diet term traditional contained fats and oil, high-fat meats and sugar was associated with a decreased in birth weight [49]. In Denmark another study showed that infants of women who consumed processed meats, potatoes and high diary fats with low intake of fruits and vegetables had lowest birth weights [52] while in New Zealand women who consume traditional diet were less likely to give birth to SGA infant [19]. Beside this, In America researchers found that WAZ and HCZ were significantly lower in infants of mothers who consume inappropriate diet compared to infants of mothers have ‘healthy dietary’ patterns [20]. On the whole, there appeared to be consistence across all studies with better outcomes in terms of birth weight with what are described as healthier diets as observed in this study.

Beside this, quality of diet among women in developing countries has always been a challenge especially during pregnancy. Researchers have attributed this to imbalance macronutrients consumption, inadequate micronutrient intake and predominantly plant-based diet [16, 47]. Similar observation was made in this study. The women dietary diversity score (a proxy of micronutrient of individual women), which ranges between 0–9, was low especially among mothers who gave birth to low birth weight infants. Other studies have also shown that maternal dietary diversity is a predictor of mean birth weight and low birth weight [27], which is consistent with our findings.

It is important to state that apart from maternal nutrition, there are several other factors that could influence infant weight at birth. The most important ones include smoking, alcohol consumption, malaria and HIV infections. None of the study participants in the present study smoked, consumed alcohol or was HIV positive. Besides this, prophylactic drugs such as Sulfadoxine-pyrimethamine are administered to all pregnant women attending antenatal care clinics in Ghana and so malaria prevalence among the women in the present study was not evident.

It is observed that there are weaknesses to using the posteriori method to determine dietary patterns since dietary patterns are not chosen in advance but are determined by the data. As a result of this two studies cannot identify identical diets thus making direct comparison between different studies impossible [19]. Beside this, there are no known established cut-offs standards for additional percentage of variance explained to decide on the number of factors that should be retained or on the cut-off for loading scores to decide on which foods should be considered relevant in every pattern identified^19^. However, to date the results generated from this kind of analysis according to researchers have shown logical relationships of dietary patterns to socio-economic factors and to outcomes such as birth weight as observed in this study. This reinforces the suitability of using this type of analysis to analyze data on diet.

Also fasting during pregnancy could affect dietary intake as well as fetal growth as some of the women might have fasted in the month of Ramadan. However, there were not questions on whether the mothers fasted or not during the month of Ramadan which, is a set back to the current study because the effect of fasting in the month of Ramadan was not ascertained and for that matter the result presented here should be interpreted with caution.

Another limitation was that information on pregnancy dietary intakes was taken retrospectively. As a result, recall bias could be inevitable, especially for mothers who just delivered but we did, however, probe or give cues to the mother about the period in question, to help recall.

Besides this, the researchers tried to make the food list as comprehensive as possible but this not withstanding, some other lesser-consumed foods might have been left out. For example some wild fruits and vegetables, which are seasonal could have been left out but this was unlikely to alter the patterns identified in the current study since they are less frequently consumed.

## Conclusion

Poor dietary practices during pregnancy such as ‘pica’ and ‘out of home eating’ were found to be associated with increase odds for low birth weight. We also identified two dietary patterns among pregnant women in Northern Ghana. These are Health conscious diet and non-health conscious diet. Women dietary diversity score and dietary patterns were found to be protective against low birth weight. Our findings buttress the importance of optimal nutrition during pregnancy. Therefore, it is important that emphasis should be placed on counseling and assisting pregnant women and prospective mothers to practice optimal nutrition through the consumption of foods across the various food groups that are good sources of both macro and micronutrients during nutrition counseling in antenatal clinics more so in Ghana.

## Supporting Information

S1 Dataset Data from maternal and child health survey, STATA format. (DTA)
